# The Association Between Medicare Annual Wellness Visits and Detection and Management of Diabetes Among Older Adults

**DOI:** 10.1093/geroni/igab046.2353

**Published:** 2021-12-17

**Authors:** Kara Dassel, Abdulrahman Alsulami, Yao He, Nancy Allen

**Affiliations:** 1 University of Utah, Salt Lake City, Utah, United States; 2 University of Utah, Jeddah, Makkah, Saudi Arabia; 3 University of Utah, Salt Lake, Utah, United States

## Abstract

The rising prevalence of diabetes mellitus (DM) among older adults is an increasing concern in the U.S. and is expected to nearly triple within the next 40 years. The purpose of this study is to investigate the effectiveness of Medicare Annual Wellness Visits (AWV) utilization on the management of DM among Medicare beneficiaries using data from 26,703 Medicare beneficiaries seen at 13 primary care community clinics (clinic visits between 2017 and 2019). A total of 34% of Medicare beneficiaries participated in an AWV. The total sample was, on average, 72.6 years old (SD=7.0), 57% female, 84% White, and 91% non-Hispanic and had between zero and three co-morbid conditions. The AWV group was significantly younger (mean difference 2.0 years; p<.001) and had fewer comorbid conditions (mean difference 0.1; p<.001) than the non-AWV group at their initial visits. Comparing AWV and non-AWV groups at the first patient visit and last patient visit, there were significantly fewer patients with DM in the AWV group compared to the non-AWV groups (19.2% vs. 24.7%; p<.001 and 53.5% vs. 59.2%; p<.001). DM management was better in the AWV group compared to the non-AWV group at both the first and last patient visits, as exhibited by lower A1C levels (M= 5.9(SD=0.8) vs. M=6.2(SD=1.1); p<.001 and M= 6.6(SD=0.8) vs. M=6.9(SD=1.4); p=.013), lower glucose levels (M=114.0(SD=34.0) vs. M=123.0(SD=51.0); p<.001), and fewer DM medications (M=0.1(SD=.4) vs. M=0.2(SD=0.5); p<.001 and M=0.2(SD=0.6) vs. M=0.3(SD=0.6); p<.001). These results suggest that AWV are effective managing diabetes in older adults Medicare beneficiaries.

